# Utility of blood DNA methylation and immune cell-type estimates to identify clinically relevant links between immunity and health conditions in a large cohort

**DOI:** 10.3389/fimmu.2025.1613557

**Published:** 2025-12-10

**Authors:** Marisol Herrera-Rivero, Jan Homann, Christina M. Lill, Matthias Nauck, Wolfgang Lieb, Jens Kuhle, Heike Minnerup, Marco Hermesdorf, Klaus Berger

**Affiliations:** 1 Institute of Epidemiology and Social Medicine, University of Münster, Münster, Germany; 2 Joint Institute for Individualization in a Changing Environment (JICE), University of Münster and Bielefeld University, Münster, Germany; 3 Ageing Epidemiology Unit, School of Public Health, Imperial College London, London, United Kingdom; 4 Institute of Clinical Chemistry and Laboratory Medicine, University Medicine Greifswald, Greifswald, Germany; 5 German Centre for Cardiovascular Research (DZHK), Partner Site Greifswald, Greifswald, Germany; 6 Institute of Epidemiology, Christian-Albrechts-University of Kiel, Kiel, Germany; 7 Multiple Sclerosis Centre and Research Center for Clinical Neuroimmunology and Neuroscience (RC2NB), Departments of Biomedicine and Clinical Research, University Hospital and University of Basel, Basel, Switzerland; 8 Department of Neurology, University Hospital and University of Basel, Basel, Switzerland

**Keywords:** DNA methylation, inflammation, granulocytes, C-reactive protein, chronic pain

## Abstract

The immune system plays a central role in health and disease. Changes in the proportions of immune cell types have been linked with diverse pathological conditions. However, direct measurements of circulating immune cells are often not available, especially in large human cohorts. Here, whole blood DNA methylation (DNAm) array data might be a feasible solution to study the links between immune cells and human health. This study investigated associations between DNAm estimates of six immune cell types and fifteen circulating serum biomarkers related to health conditions among 1007 participants of the BiDirect Study and then performed follow-up analyses. We found an association between the granulocyte estimates (Gran) and serum levels of high-sensitivity C-reactive protein (hsCRP; beta=0.021, p=3.71x10^-5^, false discovery rate-FDR=0.0038). This was further confirmed by polygenic score analysis (beta=0.0325, p=0.041). In follow-up analyses, we identified a statistical interaction between Gran and hsCRP linked to the diagnosis of chronic pain (beta = -5.65, p=0.0047, FDR = 0.047) and showed significant Gran-hsCRP interaction effects on pain perception as assessed with the pain sensitivity questionnaire (PSQ-minor; beta=0.43, p=0.0125). In addition, by analyzing DNAm levels in granulocyte-expressed genes, we pinpointed potential novel target genes for chronic pain, including *PLBD1* and *TAPBP*. These findings exemplify the utility of blood DNAm and immune cell-type estimates to help identify clinically relevant links between immunity and health outcomes in large cohorts.

## 1 Introduction

The immune system plays a central role in health and disease. As different immune cell types have different roles, their distribution in the body determines different immune functions (1). Changes in the proportions of various immune cell types have been linked with pathological conditions, including somatic and psychiatric diseases, as well as with the exposure to disease risk factors (2, 3). Therefore, shifts in immune cell-type proportions might represent clinically relevant predictors for risk assessment, diagnosis and prognosis in the context of an array of physiological and mental health conditions. However, because these changes might be subtle in non-immune disorders, measurements of circulating immune cell counts are rarely available in human cohorts outside of this context. In such cases, a feasible and pragmatic alternative is presented by the advent of DNA methylation (DNAm) profiling in whole blood. Due to the high cell-type specificity of DNAm, current deconvolution algorithms can render accurate quantifications of cell-type fractions using DNAm array data. Such estimates have proven useful resources to interrogate the links between immune cell-type shifts and diverse phenotypes in large samples (3).

In the current study, we aimed to assess the utility of whole blood DNAm and derived estimates of primary immune cell types to explore the relationships between immune variability and health in the German BiDirect Study. To achieve this, we performed a screening analysis where we tested the associations between six cell types and fifteen health-related biomarkers. Next, we conducted data-driven analyses to follow-up on significant findings and explore potential implications for disease phenotypes. This approach revealed links between granulocytes, C-reactive protein (CRP) and chronic pain in BiDirect.

## 2 Methods

### 2.1 Study population

The study design and methods of the BiDirect Study have been described elsewhere (4). Briefly, the study recruited over 2300 participants in the district of Münster, Germany, that were allocated to one of three cohorts: 1) a depression cohort (patients hospitalized due to an acute depressive episode at the time of recruitment, n=1004), 2) a cardiovascular cohort (patients with an acute coronary event three months before recruitment, n=348), or 3) a population-based cohort (randomly sampled community-dwelling adults, n=965). BiDirect participants underwent extensive phenotyping within a 10-year span, including four examinations with face-to-face interviews, laboratory tests, neuropsychological assessments, genotyping and DNAm profiling, and self-administered questionnaires applied during examinations and intermediate follow-ups. The current investigation employed a subsample of 1007 BiDirect participants from the depression (cohort 1, n=442) and population-based (cohort 3, n=565) cohorts with DNAm data available from the baseline assessment (mean age: 53 ± 8; females: 536, 53%).

### 2.2 Immune cell-type estimates

DNAm levels were measured from whole blood samples in two batches using the Infinium MethylationEPIC v1.0 or v2.0 BeadChip (Illumina Inc. San Diego, USA) arrays. Cell-type estimations were made from raw DNAm beta-values for granulocytes (Gran), monocytes (Mono), B cells (Bcell), CD4^+^ T cells (CD4T), CD8^+^ T cells (CD8T) and natural killer (NK) cells according to the method by Houseman et al., 2012 (5) using the online DNA Methylation Age Calculator (https://dnamage.clockfoundation.org/) (6).

### 2.3 Health-related biomarkers

Fifteen laboratory-derived blood measurements were tested for association with the immune cell-type estimates. These included metals (iron, copper, zinc), markers of inflammation (high-sensitivity C-reactive protein [hsCRP], interleukin [IL]-6, IL-1beta, tumor necrosis factor [TNF]-alpha and interferon [IFN]-gamma), liver function (alanine aminotransferase [ALAT] and aspartate aminotransferase [ASAT]), thyroid function (free tri-iodothyronine [fT3], free thyroxine [fT4] and thyroid stimulating hormone [TSH]) and neurodegeneration (neurofilament light chain [NfL] and glial fibrillary acidic protein [GFAP]).

### 2.4 First-step follow-up

#### 2.4.1 Confirmatory polygenic score analysis

Follow-up analyses of the identified immune cell-biomarker associations used polygenic scores (PGSs) to elucidate the relationship between DNAm estimates and the genetic determinants of immune cells and seek genetic support for the identified links with biomarkers. PGS weight files were obtained from the PGS Catalog (7) for the proportions of lymphocytes (Cat.ID: PGS003941), monocytes (Cat.ID: PGS003942), neutrophils (Cat.ID: PGS003943), eosinophils (Cat.ID: PGS003944) and basophils (Cat.ID: PGS003945), and for CRP (Cat.ID: PGS002164). PGSs were then calculated in BiDirect by applying allelic scoring with plink 1.9 (8). Genotyping, quality control (QC) and genotype imputation procedures in BiDirect have been described elsewhere (9). Genotype data was available for all individuals included in this study.

#### 2.4.2 Implications for disease outcomes

To evaluate implications of the identified immune cell-biomarker associations for disease outcomes, we performed interaction analyses using data on (self-reported) lifetime physician’s diagnosis of the following diseases: depression (n_cases_=543), anxiety (n_cases_=256), addiction (n_cases_=51), psychiatric disorder (other than depression and anxiety, n_cases_=99), chronic pain (e.g. pain in back, legs or joints, migraine, fibromyalgia; n_cases_=311), heart problems (e.g. heart failure, n_cases_=117), hypertension (n_cases_=522), kidney disease (n_cases_=83), diabetes (n_cases_=87) and thyroid disease (n_cases_=315).

### 2.5 Second-step follow-up

#### 2.5.1 Pain scales

Follow-up analyses of the identified association with disease outcomes used data on pain scales. In BiDirect, pain perception was measured using the pain sensitivity questionnaire (PSQ-minor). Pressure pain threshold (PPT) was measured using a handheld mechanic pressure algometer (upper limit=10) on the left and right hands (index finger pad) separately. Detailed procedures are described elsewhere (10).

#### 2.5.2 DNAm analysis in cell-type gene sets

To test associations with DNAm levels of genes expressed in neutrophils and/or granulocytes, the “Granulocyte” and “Neutrophil” gene sets were obtained from Harmonizome 3.0 (11). These contained 43 and 31 genes, respectively, from which 31 overlapped between both gene sets. DNAm probes corresponding to 31 neutrophil-expressed genes, 12 non-neutrophil granulocyte-expressed genes and CRP were identified using the array’s annotation file. Data pre-processing and QC followed standard procedures as previously described (12). Briefly, these included 1) data conversion, 2) calculation of detection p-values, 3) removal of probes that failed detection, located on the sex chromosomes, contained polymorphisms, and/or are cross-reactive, 4) removal of outlier samples, 5) calculation of normalized beta values using the dasen method, and 6) transformation into M-values for use in statistical analyses. These procedures were performed using the bigmelon (13) R package. The M-values of 265 probes annotated to selected genes and available in both BiDirect batches after QC were used for statistical analysis.

### 2.6 Statistical analysis

Cell-type estimates, laboratory measurements and pain scores were log-transformed to approximate normality. In all instances, individuals with missing values and outliers at ±3 standard deviations were removed for both cell-type and test phenotype prior to running the test, and robust regression models (R package “robustbase” (14)) adjusting for age, sex, cohort assignment, current smoking status, years of education and body-mass index (BMI) were employed. Sensitivity analyses using the original values of cell-type estimates were conducted. For significant findings, sensitivity analyses also adjusted for batch/array version with and without adjustment for years of education. For PGS analysis, the first five genomic principal components were added to the set of covariates. In the biomarker and PGS analyses, cell-type estimates were used as the dependent variable in the regression models. Interaction tests employed logistic regression for the dichotomous disease outcomes or linear regression for the pain scales as the dependent variables and a cell-type*biomarker interaction element. For both algometer variables, tobit models for right-censored data were applied using the R package “VGAM” (15). Regression results are reported as beta (i.e. coefficient estimate), standard error (SE) and p-value. For DNAm data, the first five DNAm principal components were added to the set of covariates and the analysis was performed using the R package “limma” (16). Results were considered statistically significant at false discovery rate (FDR)<0.05 in the screening analyses (i.e. association with laboratory measurements and interaction tests for disease outcomes). All other tests were considered significant at the nominal threshold (p<0.05).

## 3 Results

**Figure 1:**
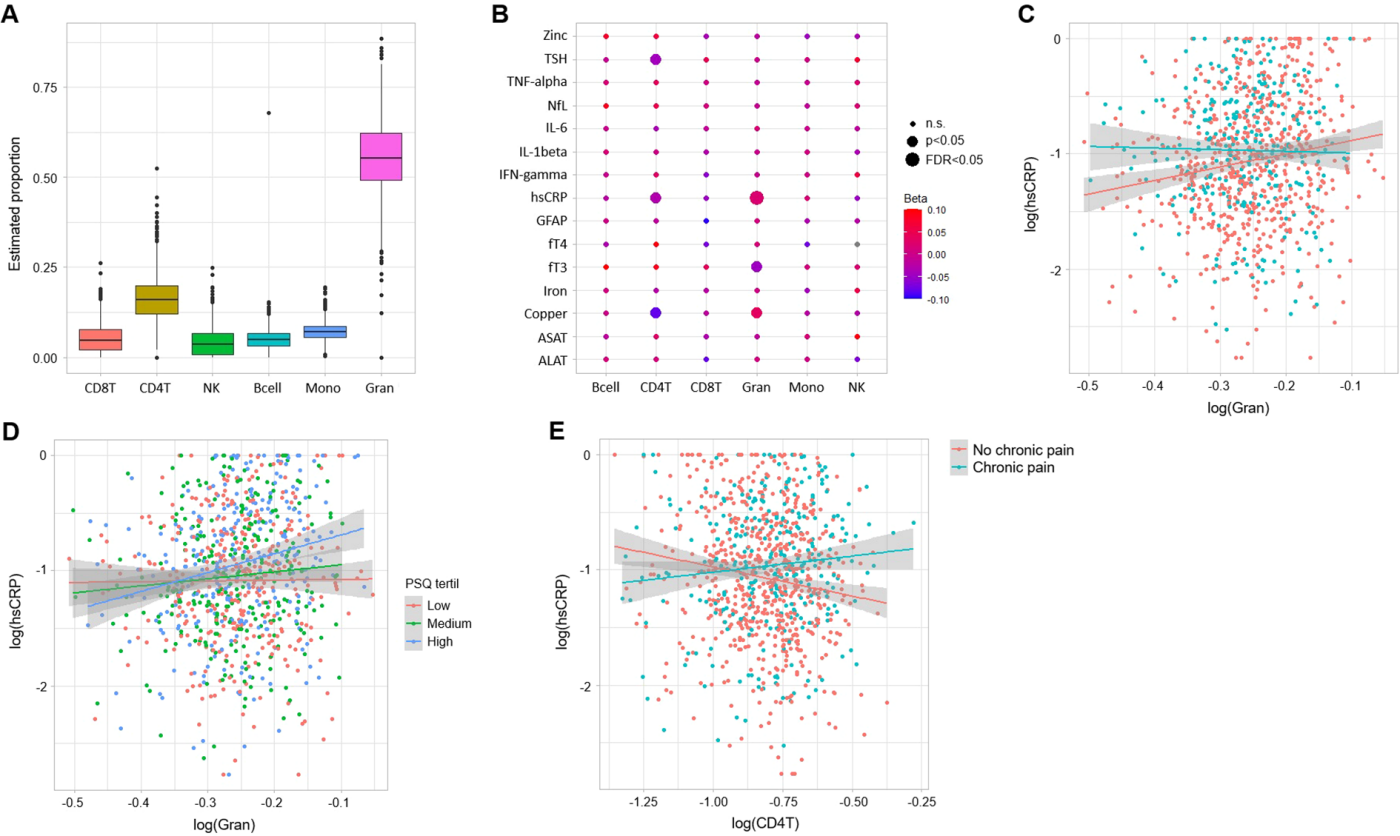
**(A)** DNAm-derived immune cell-type estimates (raw values representing relative proportions). **(B)** Results from the association analysis between DNAm-estimated immune cell-type proportions and health related biomarkers measured in serum/plasma. Coefficient estimates (Beta) are shown in color gradient. Significance levels are depicted by size; n.s.: non-significant. **(C)** Interaction between the proportion of granulocytes and hsCRP levels in chronic pain. The plot shows log-transformed values of Gran and hsCRP, stratified by chronic pain diagnosis. **(D)** Interaction between the proportion of granulocytes and hsCRP levels in pain perception. The plot shows log-transformed values of Gran and hsCRP, stratified by the level of PSQ-minor (categorized as low, medium and high according to the calculated tertils). **(E)** Interaction between the proportion of CD4^+^ T cells and hsCRP levels in chronic pain. The plot shows log-transformed values of CD4T and hsCRP, stratified by chronic pain diagnosis.

The highest and lowest cell-type estimates were found for Gran and NK, respectively (**Figure 1A**). After excluding individuals with missing data and outliers, the effective sample sizes for each association test in the initial screening phase ranged between 702 and 963 individuals. Results from this analysis showed a statistically significant association between Gran and hsCRP (beta=0.021, SE = 0.005, p=3.7x10^-5^, FDR = 0.0038; n=931). Follow-up PGS analysis confirmed strong associations of Gran with the PGSs for the proportion of neutrophils (beta=0.1, SE = 0.017, p=6.72x10^-9^) and lymphocytes (beta = -0.089, SE = 0.017, p=3.81x10^-7^), and modest associations with the PGSs for the proportion of monocytes (beta = -0.039, SE = 0.019, p=0.0382) and CRP (beta=0.0325, SE = 0.016, p=0.041). Although only the association between Gran and hsCRP remained significant after correction for multiple comparisons in the initial screening analysis, nominal associations (p<0.05) were observed for Gran with fT3 and copper, for CD4T with hsCRP, copper, zinc and TSH, and for CD8T with hsCRP. Results from this screening analysis are shown in **Figure 1B**.

Next, we investigated whether the association between Gran and hsCRP might be clinically relevant by conducting analyses that tested interaction effects of Gran and hsCRP on disease diagnoses. This analysis identified a significant interaction between Gran and hsCRP in the context of lifetime chronic pain diagnoses (beta = -5.65, SE = 1.9, p=0.0047, FDR = 0.047; **Figure 1C**). The independent effects on chronic pain diagnosis of both Gran (beta = -7.9, SE = 2.3, p=7x10^-4^) and hsCRP (beta = -1.36, SE = 0.5, p=0.0094) were also significant. Sensitivity analyses showed robustness of this finding. Also, about 32% of the study sample had history of a physician’s diagnosis of (general) chronic pain by the end of the BiDirect Study, with 219 individuals reporting this at baseline examination (i.e. prevalent chronic pain) and 92 reporting it afterwards (i.e. incident chronic pain). Therefore, we considered this relationship to be a valid observation and conducted further analyses.

Following-up on the Gran-hsCRP link to chronic pain, we found that the Gran-hsCRP interaction was slightly more significant for incident (p=0.017) than prevalent (p=0.029) chronic pain diagnoses. However, given that the relationship was significant in both instances, with effects following the same direction, we chose to continue using unstratified analyses. First, we tested the effects of Gran and hsCRP on pain sensitivity (PPT) and perception (PSQ-minor). All pain measures tested were associated with chronic pain (negative estimates for sensitivity and positive for perception). However, the Gran-hsCRP interaction was only significant for the PSQ (beta=0.43, SE = 0.17, p=0.0125; **Figure 1D**). The independent effects of Gran (beta=0.49, SE = 0.22, p=0.023) and hsCRP (beta=0.14, SE = 0.05, p=0.0023) on PSQ measures were significant. In addition, because CD4T and CD8T showed nominally significant associations with hsCRP, we also tested these interactions in chronic pain and found that CD4T, but not CD8T, positively interacted with hsCRP (beta=2.5, p=0.00175; n=820; **Figure 1E**) in chronic pain. However, CD4T showed no interaction effects with hsCRP on pain sensitivity or perception. Second, we explored associations of chronic pain diagnosis and hsCRP with DNAm levels in granulocyte-expressed genes and the CRP gene. Results of this analysis are shown in **Table 1**. After correction for multiple comparisons, we found that two methylation probes (cg22488164 in *PLBD1* and cg24777950 in *CTSG*) were associated with hsCRP (n=926), while none remained significant after correction for multiple comparisons in the chronic pain analysis (n=961, 318 cases and 643 controls). Nominal (p=0.05) findings included another eight probes corresponding to six genes in the hsCRP analysis, and 12 probes corresponding to seven genes in the chronic pain analysis. Both analyses showed the same top probe (cg22488164 in *PLBD1*). Another three overlapping genes (*GCH1*, *PDGFRA* and *TAPBP*) coming from different probes were observed within the nominal-level findings.

**Table 1:** Nominal-level findings from the gene set DNAm analysis.

ProbeID	Gene	GeneSet	Chr	Position	Annotation(s)	Beta	p-value
hsCRP							
cg22488164	***PLBD1***	**Neutrophils**	**12**	**14716910**	**Body**	**0.0643**	**1.83E-05**
cg24777950	*CTSG*	Neutrophils	14	25046121	TSS1500	-0.0378	2.58E-04
cg23357789	*PRTN3*	Neutrophils	19	848026	3’UTR	-0.0357	1.16E-02
cg25141766	*ARHGDIA*	Neutrophils	17	79826316	3’UTR; enhancer	0.0444	1.37E-02
cg20734421	*PLBD1*	Neutrophils	12	14720805	TSS200	0.0449	1.79E-02
cg14039343	*TAPBP*	Neutrophils	6	33267684	3’UTR; promoter-associated	-0.0216	2.16E-02
cg26681354	*GCH1*	Granulocytes	14	55369212	1stExon; promoter-associated	0.0251	0.03381
cg20379685	*PDGFRA*	Granulocytes	4	55125798	Body; enhancer	0.0315	0.03726
cg01963696	*ELANE*	Neutrophils	19	851650	TSS1500	0.0228	0.03743
cg06406619	*ELANE*	Neutrophils	19	851045	TSS1500; enhancer	-0.0266	0.04401
Chronic pain							
cg22488164	***PLBD1***	**Neutrophils**	**12**	**14716910**	**Body**	**0.0463**	**3.95E-03**
cg24898863	*S100A8*	Neutrophils	1	153363580	TSS200	-0.0305	4.11E-03
cg02058516	*PDGFRA*	Granulocytes	4	55142142	Body; enhancer	0.0469	6.08E-03
cg23922433	*TAPBP*	Neutrophils	6	33279563	Body	-0.0288	0.01743
cg00708486	*GCH1*	Granulocytes	14	55355071	Body; enhancer	-0.0592	0.02277
cg14006309	*TAPBP*	Neutrophils	6	33281552	Body; promoter-associated	-0.0331	0.02339
cg22878148	*PDGFRA*	Granulocytes	4	55104084	5’UTR	-0.0298	0.02664
cg22727297	*TAPBP*	Neutrophils	6	33272371	Body	-0.0258	0.03152
cg06751597	*SNAP23*	Neutrophils	15	42788175	5’UTR; promoter-associated	0.0374	0.03342
cg14039343	*TAPBP*	Neutrophils	6	33267684	3’UTR; promoter-associated	-0.0213	0.03609
cg16995420	*TAPBP*	Neutrophils	6	33282018	5’UTR; promoter-associated	-0.0299	0.04344
cg16987556	*FIP1L1*	Granulocytes	4	54245040	Body; promoter-associated (cell-type specific)	-0.0468	0.04563

The first row in each of the table is in bold font, meaning "Both analyses had the same top probe".

## 4 Discussion

This study found associations of DNAm granulocyte estimates with CRP levels and CRP-PGS, as well as an interaction between Gran and CRP that is relevant for pain phenotypes. The finding that Gran showed a strong positive association with the genomic determinants of neutrophil proportion of leukocytes suggests that, in agreement with the literature, the major constituent of Gran are neutrophils, the most abundant type of granulocytes in the human body (1). It is known that CRP contributes to toxic granulation in neutrophils (17). Neutrophils are key in chronic inflammation (18) and chronic widespread pain in fibromyalgia (19). Moreover, higher CRP levels in individuals with chronic pain and higher pain sensitivity have been previously reported (20–22). These observations validate the utility of DNAm-derived immune cell-type estimates to study relationships between immunity and disease phenotypes in large cohort studies such as BiDirect. Beyond this, our study provides evidence of a significant interaction between immune cell proportions and CRP in the context of pain phenotypes which, to our knowledge, has not been reported before. This interaction was observed in both prevalent and incident chronic pain diagnoses in the BiDirect Study, suggesting an involvement in the development of chronic pain and potential for risk assessment. Of note, even though the diagnosis of chronic pain was associated with both medical and psychiatric diagnoses, including depression, anxiety, addiction, and heart and kidney disease, the Gran-CRP interaction was not associated with these other diagnoses, suggesting a direct relationship with pain phenotypes.

Associations with CRP were also suggested for CD4^+^ and CD8^+^ T cell estimates derived from DNAm data in our study. Although CRP is well-known for its role in inflammation, contributions to adaptive immunity via binding of CD4^+^ T cell subsets have been proposed (23). The evidence of association between CD4T and CRP was strengthened by our observation of a significant interaction of these variables in chronic pain in opposite direction to that found for Gran. Although a number of past studies found no changes in T cell counts in individuals with chronic pain, it is believed that specific CD4^+^ T cell subsets might regulate the transition from acute to chronic pain (24). In addition, the strong negative relationship between Gran and the PGS for lymphocytes in our study is in accordance with previous knowledge, as the neutrophil-to-lymphocyte ratio (NLR) has been used as a marker of inflammation involved in several diseases and overall mortality (25).

Finally, DNAm analysis identified methylation sites in *PLBD1* and *CTSG* associated with CRP levels. Both genes have been previously linked to pain phenotypes (26, 27). Although our DNAm analysis did not reveal strong relationships between DNAm levels in granulocyte genes and a chronic pain diagnosis, nominal findings highlighted specific genes that might be relevant for pain phenotypes and worth of further investigation. In particular, *PDGFRA* (28), *GCH1* (29, 30) and *S100A8* (31, 32) have been previously associated with pain in different contexts and are targets of approved drugs. Similarly, although DNAm analyses stratified by prevalent or incident chronic pain were fairly underpowered and, therefore, results from these were not shown here, it is worth noting that the most significant DNAm probes for incident chronic pain located in promoter regions of *SNAP23* (cg26348910, p=0.0045, effect=0.08; cg14205524, p=0.01, effect=0.074), whose protein product has been linked to neuropathic pain (33). Therefore, this gene might be an interesting candidate for follow-up studies that investigate its role and potential utility as predictive marker for the development of chronic pain.

Our study has some limitations. First, DNAm data was generated only for a fraction of BiDirect participants, limiting the sample size accessible for this investigation. Second, in order to avoid high burden of multiple testing, we limited the number of variables tested during the initial screening analysis. Nevertheless, our aim was not to conduct a phenome-wide analysis. Instead, we were interested in utilizing representative blood biomarkers associated with health status for this initial phase of our study. Third, chronic pain is broadly conceptualized here. However, we believe that this broad conceptualization might represent an advantage if we consider the importance of understanding the general mechanisms underlying the development of chronic pain phenotypes. Fourth, our study showed low power to detect associations with DNAm levels. Even if the literature seems encouraging, more research will be needed to determine if and how the suggested genes might be involved in the pathobiology of chronic pain and could be targeted for the management of this condition.

Overall, we found that individuals with chronic pain in the BiDirect Study showed higher pain perception but lower sensitivity to pain, as well as a shift in the relationships between serum CRP and the proportions of granulocytes and CD4^+^ T cells. Lower Gran levels appeared to add to the effects of higher CRP levels on increasing pain perception without affecting pain sensitivity, while CD4T showed no combined effects with CRP on pain ratings. Moreover, DNAm in specific granulocyte-expressed genes appeared to correlate with both CRP and chronic pain. These observations may have translational value for the management of pain phenotypes, demonstrating the utility of DNAm and derived cell-type estimates to identify relevant associations between immunity and pathological conditions in the BiDirect Study and, more generally, in large human cohorts.

## Data Availability

The original contributions presented in the study are included in the article/Supplementary Material. Further inquiries can be directed to the corresponding author.
